# The ergogenic effect of acute carnosine and anserine supplementation: dosing, timing, and underlying mechanism

**DOI:** 10.1080/15502783.2022.2053300

**Published:** 2022-03-26

**Authors:** Sarah de Jager, Laura Blancquaert, Thibaux Van der Stede, Eline Lievens, Siegrid De Baere, Siska Croubels, Ettore Gilardoni, Luca G. Regazzoni, Giancarlo Aldini, Jan G. Bourgois, Wim Derave

**Affiliations:** a Department of Movement and Sports Sciences, Ghent University, Ghent, Belgium; b Department of Pharmacology, Toxicology and Biochemistry, Ghent University, Merelbeke, Belgium; c Department of Pharmaceutical Sciences, University of Milan, Milan, Italy

**Keywords:** Carnosine, anserine, ergogenic supplement, neuromuscular function, muscle perfusion

## Abstract

**Background:**

Recent studies suggest that acute-combined carnosine and anserine supplementation has the potential to improve the performance of certain cycling protocols. Yet, data on optimal dose, timing of ingestion, effective exercise range, and mode of action are lacking. Three studies were conducted to establish dosing and timing guidelines concerning carnosine and anserine intake and to unravel the mechanism underlying the ergogenic effects.

**Methods:**

First, a dose response study A was conducted in which 11 men randomly received placebo, 10, 20, or 30 mg.kg^−1^ of both carnosine and anserine. They performed 3x maximal voluntary isometric contractions (MVC), followed by a 5 x 6 s repeated cycling sprint ability test (RSA), once before the supplement and 30 and 60 minutes after. In a second study, 15 men performed 3x MVCs with femoral nerve electrical stimulation, followed by an RSA test, once before 30 mg.kg^−1^ carnosine and anserine and 60 minutes after. Finally, in study C, eight men performed a high intensity cycling training after randomly ingesting 30 mg.kg^−1^ of carnosine and anserine, a placebo or antihistamines (reduce post-exercise blood flow) to investigate effects on muscle perfusion.

**Results:**

Study A showed a 3% peak power (p = 0.0005; 95% CI = 0.07 to 0.27; ES = 0.91) and 4.5% peak torque (p = 0.0006; 95% CI = 0.12 to 0.50; ES = 0.87) improvement on RSA and MVC, with 30 mg.kg^−1^ carnosine + anserine ingestion 60 minutes before the performance yielding the best results. Study B found no performance improvement on group level; however, a negative correlation (r = −0.54; p = 0.0053; 95% CI = −0.77 to −0.19) was found between carnosinase enzyme activity (responsible for carnosine and anserine breakdown) and performance improvement. No effect of the supplement on neuromuscular function nor on muscle perfusion was found.

**Conclusions:**

These studies reveal that acute ingestion of 30 mg.kg^−1^ of both carnosine and anserine, 60 minutes before a high intensity exercise, can potentially improve performance, such as short cycling sprints or maximal muscle contractions. Subjects with lower carnosinase activity, and thus a slower breakdown of circulating dipeptides, appear to benefit more from this ergogenic effect. Finally, neither the involvement of a direct effect on neuromuscular function, nor an indirect effect on recovery through increased muscle perfusion could be confirmed as potential mechanism of action. The ergogenic mechanism therefore remains elusive.

## 1. BACKGROUND

Carnosine (beta-alanyl-L-histidine; CAR) and its methylated analog anserine (beta-alanyl-N(pi)-methyl-L-histidine; ANS) are histidine-containing dipeptides (HCD) abundantly present in mammalian skeletal muscle, and hence in the daily diet of omnivorous humans. Throughout the years, both dipeptides have been extensively studied and were shown to have various health [1] and performance-related properties [2,3].

Over 60 years ago, a Russian scientist, Sergei Severin, showed for the first time that acute HCD supplementation could have a performance enhancing effect [4]. Adding CAR to a neuromuscular preparation of a frog Sartorius muscle resulted in increased muscle force and a delay in time to exhaustion [4–6]. This was the first indication that acute supplementation with HCD could have a performance enhancing effect. In 2014, Baguet and colleagues attempted to translate the Severin’s phenomenon to humans. Subjects ingested 20 mg.kg^−1^ body mass (BM) of CAR 35 minutes before starting a supramaximal cycling test, however no performance improvement was found. Notably, only three of the 12 subjects in this study showed a detectable and measurable plasma CAR concentration 50 min after ingestion of the supplement, which is hypothesized as a possible explanation for the lack of performance improvement [7]. The reason for these low to undetectable CAR concentrations is the presence of a highly active, HCD hydrolyzing, enzyme in human circulation, namely serum carnosinase (CN1). It is known that the affinity of CN1 toward ANS is, in comparison to CAR, lower due to the methylation of the imidazole-ring, resulting in a delay in breakdown of the dipeptide upon oral ingestion [8–12]. Indeed, an in vivo pharmacokinetic experiment showed that the combined ingestion of both dipeptides increases plasma accumulation of ANS beyond the level measured following ANS ingestion without CAR [13].

Literature on the effect of acute supplementation with combined CAR and ANS on exercise performance is scarce and somewhat controversial. Suzuki and colleagues performed two similar performance studies in which subjects acutely ingested Chicken Breast Extract (CBEX; 1.1 g ANS + 0.4 g CAR). A first study showed a positive effect at the end of a series of short cycling sprints while they were unable to replicate these results in a follow-up study [14,15]. Also, Barbaresi et al. (2021) found an overall improved mean power output during an 8 min cycling time trial after acutely ingesting a chicken broth containing 15.6 mg.kg^−1^ CAR and 30.8 mg.kg^−1^ ANS. However, the supplements used in these studies were derived from chicken meat and therefore contain other types of bioactives (which were absent in the placebo soup) such as proteins, smaller peptides, and amino acids, apart from CAR and ANS.

Recently, Blancquaert and colleagues (2021) reported two performance studies in which a balanced and chemically synthesized mixture of 25 mg.kg^−1^ of both pure CAR and ANS was administered. A 6.1% increase in peak power output during the first seconds of an all-out Wingate test after a 6 minute exhaustive cycling protocol as well as a 2.5% improved peak power during a repeated Wingate protocol (3x 30 s Wingate with 4 min rest in between) was observed. Although several studies were able to elicit performance improvements with acute CAR and ANS supplementation, it remains unclear what the most effective dose and circulating plasma HCD concentration is to evoke these ergogenic effects.

Despite the observed improvements in performance in several studies [13,15,16], no differences were found in plasma lactate concentration, pH, bicarbonate concentration, and glutathione as oxidative stress marker. This indicates that the benefits currently can not be attributed to the buffering or antioxidant effect of HCD, which are usually described as their main functions [17]. In order to optimize and effectively implement the supplement in a sports performance setting, it is necessary to understand the mechanism underlying the ergogenic effects. An interesting finding by Nagai and colleagues (2019) might be a new lead to explore. They found that in vivo topical administration of CAR onto the femoral skeletal muscle stimulates the activity of the sympathetic nerve in the contralateral leg in rats. Additionally, intraduodenal administration of CAR resulted in a 5 to 15% increase of muscle blood flow [18,19]. The current paper therefore aims to explore an effect on neuromuscular function (as originally proposed by Severin) and/or muscle hemodynamic processes as potential ergogenic mechanisms.

For this purpose, we performed three acute intervention studies in humans. First, a dose-response study (Study A) was carried out to determine potential minimal doses or plasma HCD concentrations above which performance is improved. Second, neuromuscular electrical stimulation of human quadriceps muscle was applied to explore neural or excitation-contraction coupling involvement in these effects (Study B). It was expected that subjects, after CAR and ANS ingestion, would be able to voluntarily contract muscles closer to their full potential, compared to the placebo condition. Third, Doppler Ultrasound was used to evaluate whether muscle perfusion during the recovery of a high-intensity cycling performance was affected by combined CAR and ANS ingestion (Study C). An increase in muscle perfusion following the supplement was expected.

## 2. METHODS

### General information (Study A, B, and C)

This paper includes three human performance studies. All subjects were physically active males (exercise at least 3 times per week for more than 30 min), nonsmoking and free of supplement use such as beta-alanine, carnosine, and creatine (Study A, B, C) and anti-allergy medication (Study C) three months prior to and during the study. Supplements such as multi-vitamins, coffee (not allowed on test days), recovery shakes, etc. were allowed during the months prior to the study. All subjects gave their written informed consent and studies were approved by the local Ethics Committee of the Ghent University Hospital (Reference number 2014/0808). The studies were performed in accordance with the standards of ethics outlined in the Declaration of Helsinki.

### Experimental design

The studies included in this paper are three double-blind, placebo-controlled, crossover laboratory studies (Study A, B, and C) for which different subjects were recruited. For all three studies, randomization was performed following the simple randomization method to ensure a balanced division in order of conditions. This was done by a coworker who was, apart from the pill making, not further involved in the study. Test days were separated by at least 48 hours and subjects conducted their performance each test day around the same time to control for diurnal variation.

#### Study A: dose-response study

In this study, a possible dose-response relationship was determined for ergogenic effects of combined CAR and ANS ingestion.

*Subjects*. Eleven men volunteered to participate in this study. The subjects’ body mass, height, and age were 74.1 ± 4.3 kg, 180.7 ± 4.3 cm, and 22.7 ± 2.5 years, respectively. Their CN1 activity, which was determined after the study, ranged between 0.4 and 2.8 µmol.mL^−1^.hr^−1^ with an average value of 1.9 ± 0.7 µmol.mL^−1^.hr^−1^.

*Measurements*. A first performance parameter consisted of three maximal isometric voluntary contractions (MVC) of the knee extensors with the knee flexed at 90° on a dynamometer (Biodex System 3 Pro, Biodex Medical Systems). Maximal contraction was sustained for 3 s and repetitions were separated by 20 s rest intervals. A second performance was a repeated sprint ability (RSA) test consisting of 5 x 6 s all-out cycle sprints every 30s on a cycle ergometer (Cyclus 2, Avantronic). During the 24 s of active recovery between sprints, subjects were allowed to cycle at a low cadence (below 70 rpm). Short cycling sprints, rather than 30 s Wingate tests, were included due to the findings by Blancquaert et al. (2021) that the ergogenic effect is present during the initial phase of these maximal cycling tests.

Ethylenediaminetetraacetic acid (EDTA) plasma was collected to measure plasma CAR and ANS concentrations using LC-MS/MS and serum samples were collected to determine CN1 activity of the subjects, using ELISA. The exact procedure of these analytical techniques is described further.

*Supplementation*. The supplement consisted of 10 mg.kg^−1^ BM (10 mg.kg^−1^ CAR+ANS), 20 mg.kg^−1^ BM (20 mg.kg^−1^ CAR+ANS) or 30 mg.kg^−1^ BM (30 mg.kg^−1^ CAR+ANS) of each CAR and ANS, kindly provided by Flamma (Italy), in capsules or a similar amount of maltodextrine capsules (Placebo). This was ingested 30 and 60 min before the start of the performance.

*Procedure*. Subjects came to the laboratory on five occasions: a familiarization day and four actual test days during which the different doses of the supplement were tested in randomized order. During the first visit, participants were familiarized with the different performance exercises described above.

**Figure 1.**
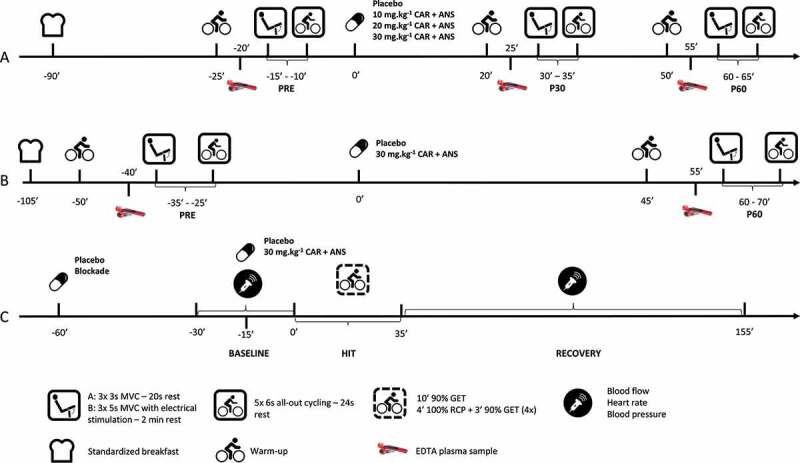
Overview of the test protocols of (A) the dose response study (= study A), (B) the study with electrical stimulation (= study B) and (C) the study with blood flow measurements (= study C).

The day before the test days, subjects were asked to refrain from exercise and they were not allowed to eat meat and fish or drink alcohol. On the actual test days, subjects arrived in a fasted state and received a standardized breakfast free of CAR and ANS (See supplemental material for a detailed description) immediately upon arrival. A standardized warm-up, consisting of 5 min of cycling at 80 W, was started 65 min after breakfast. Thereupon, the MVCs immediately followed by the RSA were performed a first time (PRE). One and a half hour after the breakfast was consumed, subjects ingested the supplement. Both 20 and 50 min following supplementation, subjects again performed the standardized warm-up for 5 min and 30 min (P30) and 60 min (P60) following supplementation the MVCs and RSA were performed a second and third time. After each warm-up, an EDTA plasma sample was collected from an antecubital vein. On the first blood withdrawal of the first test day, also a serum sample was taken to determine CN1 activity (Figure 1A).

#### Study B: placebo vs. 30 mg.kg^−1^ of both carnosine + anserine with electrical stimulation of the knee extensor muscles

In order to uncover a possible mechanism of the acute ergogenic effect of CAR and ANS supplementation, electrical stimulation (ES) of the knee extensor muscles was applied to investigate a possible effect on neuromuscular function.

*Subjects*. Fifteen men volunteered to participate in this study. Their age, body mass, and height were 23.2 ± 2.4 years old, 75.7 ± 6.7 kg and 179.3 ± 6.6 cm, respectively. The subjects’ CN1 activity, which was determined after the study, ranged between 0.6 and 2.8 µmol.mL^−1^.hr^−1^ and was on average 2.2 ± 0.7 µmol.mL^−1^.hr^−1^.

*Measurements*. The performance protocol consisted of three MVCs with supramaximal ES of the knee extensors on a dynamometer (Biodex System 3 Pro, Biodex Medical Systems), followed by the RSA (Cyclus 2, Avantronic) as described in study A.

The MVCs lasted 5 s with 2 min of rest in between. The electrical stimulation protocol was based on an existing protocol [20,21]. To perform the MVC, the subjects were instructed to reach their maximal force as fast as possible and to hold this plateau of which they received visual feedback (only the shape of the curve was shown, no quantitative data could be seen by the subjects nor the researchers). During the plateau, a superimposed doublet at a frequency of 100 Hz was administered. After the MVC, the subjects were asked to relax their muscles and three additional potentiated stimuli were given: a 100 Hz doublet, a 10 Hz doublet and a singlet. All stimuli were spaced by 2 s relaxation intervals. A single stimulus lasted 200 µs and was administered via a constant current stimulator (DS7AH, Digitimer, Ltd., Welwyn Garden City, UK). During the MVCs, electromyographical (EMG) data was collected in the vastus lateralis of the quadriceps muscle. Self-adhesive surface electrodes (Ag-AgCl, 10-mm diameter) were positioned lengthwise over the middle part of the muscle belly with an interelectrode (center to center) distance of 2 cm, according to SENIAM guidelines [22]. These electrodes were connected with wireless transmitters to the Noraxon device (3 kHz, ZeroWire; Noraxon). To determine the electrical stimulation intensity to fully contract the knee extensors, the following procedure was used: the cathode was placed over the nerve in the femoral triangle and the anode on the trochanter major of the right leg. M wave amplitude was measured in the vastus lateralis of the quadriceps femoris muscle using EMG. The intensity of the stimulation was gradually increased by 5 mA until a stronger stimulus no longer increased the amplitude of the twitch force and the peak-to-peak amplitude of the knee-extensor M waves. To ensure supramaximal stimulation, 120% of this intensity was used for the actual MVCs. The supramaximal intensity ranged between 102 and 204 mA and was constant throughout the test days.

EDTA plasma was collected to measure plasma CAR and ANS concentrations using LC-MS/MS and serum samples were collected to determine CN1 activity of the subjects using ELISA.

*Supplementation*. The supplement consisted of 30 mg.kg^−1^ BM CAR and 30 mg.kg^−1^ BM ANS (30 mg.kg^−1^ CAR+ANS), kindly provided by Flamma (Italy), or a placebo which contained maltodextrine, in capsules. This was ingested 60 minutes before the start of the actual performance.

*Procedure*. All subjects were familiarized on two occasions, separated by at least 48 hr. On both days, the intensity of electrical stimulation to attain maximal M wave amplitude and twitch force was determined using the protocol described above and the entire performance protocol, consisting of MVCs with ES and RSA, was executed.

The day before the test days, subjects were asked to refrain from exercise and they were not allowed to eat meat and fish or drink alcohol. On the actual test days, the subjects arrived in the lab after an overnight fast. The electrodes were immediately put in place and the subjects received a standardized breakfast free of CAR and ANS (See supplemental material for a detailed description). Fifty-five minutes following breakfast, the subjects started their standardized warm-up which consisted of 10 min cycling at a resistance of 100 W. After 5, 6 and 7 min, they were asked to do a 3 s sprint. Then, following the warm-up, a first round of the performance protocol (MVC with ES immediately followed by the RSA; PRE) was conducted. One hour and 45 min after breakfast, subjects ingested the supplement or placebo, in randomized order. The warm-up and performance was repeated 45 min after ingesting the supplement with the actual performance starting 60 min (P60) after ingestion. After each warm-up, an EDTA plasma sample was drawn from an antecubital vein. On the first blood withdrawal of the first test day, also a serum sample was taken to determine CN1 activity (Figure 1B).

#### Study C: the effect of 30 mg.kg^−1^ of both carnosine + anserine vs. H_1_ and H_2_ histamine receptor blockers (blockade) on recovery blood flow following a HIT

This study was conducted to investigate whether an effect on post-exercise muscle perfusion, and as a result an improved muscle recovery, could be an explanation for the ergogenic effects observed in previous performance studies.

*Subjects*. Eight men volunteered to participate in this study. Their body mass, height, and age were 71.0 ± 6.3 kg, 179.4 ± 4.4 cm, and 23.8 ± 2.0 years, respectively. The men had a mean VO_2peak_ and P_peak_ of 54.2 ± 10.6 mL/min/kg and 382.6 ± 58.1 W, respectively. The subjects’ CN1 activity, which was determined after the study, ranged between 1.2 and 3.9 µmol.mL^−1^.hr^−1^ and was on average 2.5 ± 0.9 µmol.mL^−1^.hr^−1^.

*Measurements*. A first test that was conducted was an incremental cycling test: 6 min unloaded pedaling, followed by 6 min at 110 W. Then, the subjects rested for 2 min followed by a warm-up of 4 min at 75 W, after which the incremental test started. Work rate increased with 25 W every minute until volitional exhaustion. Oxygen uptake and carbon dioxide excretion was measured breath-by-breath (Metalyzer 3B; Cortex Biophysik GmbH, Leipzig, Germany). Data were transformed into 10-s averages for further analysis. Ventilation (VE), VO_2_ and VCO_2_ data were used to determine the gas exchange threshold (GET) and respiratory compensation point (RCP). GET was visually determined using the V-slope method in combination with the first departure from the linear increase in VE, VE/VO_2_ and PETO_2_ in relation to VO_2_. RCP was determined as the second deviation from linearity in VE and the first deviation in VE/VCO_2_ and PETCO_2_. A correction for delay in VO_2_ kinetics was conducted to translate GET and RCP to corresponding power output values by calculating the mean response time and subtracting this value from the uncorrected GET and RCP. The corresponding power outputs were used to determine the intensity of the high-intensity training (HIT) during the actual test days. The HIT consisted of 10 min warm-up at 90% of the GET followed by 4 blocks of 4 min at 100% of the RCP, with 3 min active rest at 90% GET. GET and RCP were on average 202.5 ± 42.7 W and 261.5 ± 45.5 W.

Blood flow was measured in the right femoral artery using Doppler Ultrasound (Xario 100, Canon Medical Systems Europe, Zoetermeer, The Netherlands) equipped with a 11L4 linear probe at an imaging and Doppler frequency of 8.4 MHz and 4.0 MHz, respectively. The femoral artery was insonated at the lowest possible angle, always below 60°, distal to the inguinal ligament and approximately 3 cm above the bifurcation where the artery splits into the superficial and deep femoral artery. The sample volume was maximized to the artery diameter with care taken to avoid interference of the vessel walls and a standard low-velocity rejection filter was applied. Femoral artery diameter was determined in triplicate during systole with the built-in calipers [23].

Blood pressure and heart rate were measured using an automatic upper arm blood pressure measurement device (M3 Comfort, Omron Healthcare Europe B.V., Hoofddorp, The Netherlands).

*Supplementation*. The supplements that were ingested during this study consisted of: a placebo (maltodextrin), 30 mg.kg^−1^ of both CAR and ANS, ingested 15 min before the start of the HIT, and 540 mg fexofenadine (Telfast, Sanofi, Paris, France) (H_1_ receptor antagonist) and 40 mg famotidine (Aurobindo Pharma B.V., Baarn, The Netherlands) (H_2_ receptor antagonist) (Blockade condition) 1 hr before the start of the HIT. These amounts and timing were shown to adequately block both H_1_ and H_2_ receptors [23,24] and significantly blunt the post-exercise blood flow response, which is the opposite response expected following acute CAR and ANS supplementation. This extra condition was added as a negative control condition.

*Procedure*. The study consisted of four test days. During the first test day, an incremental cycling test until exhaustion was performed as described above. The following three test days went as follows: The evening before a test day, subjects were not allowed to exercise and they were asked to refrain from meat, fish, and alcohol. On the test day itself, they were asked to eat the same meal, free of meat and fish, at the same time before they came to the lab and to entirely refrain from caffeine. The subjects came to the lab 30 min before the start of the HIT and had to lie down for 30 min. Baseline measurements of muscle blood flow, heart rate, and blood pressure were measured 10 min, 5 min and immediately before the start of the exercise. For further calculations, the lowest measurement of the three baseline blood flow values was used. Then, subjects started the HIT lasting 35 min. Immediately after the last interval at 100% RCP, they were asked to lie down. Blood flow was measured at 1 min, every 5 min during the first half hour and every 10 min for the next 1.5 hr. Blood pressure and heart rate were measured twice every 10 min during the 2 hr recovery (Figure 1C).

### Plasma HCD concentration

All plasma samples were collected in pre-cooled (4°C) EDTA plasma tubes to block CN1 activity. Upon withdrawal, samples were centrifuged (5 min, 4000 g) to separate plasma and immediately deproteinized with 35% sulfosalicylic acid (SSA) in an 1:11 ratio, centrifuged (5 min, 16,000 g) and stored at −20°C awaiting further LC-MS/MS analysis.

The LC-MS/MS protocols described in Blancquaert et al. (2021) and Barbaresi et al. (2021) are referred to for a detailed description of the methods used to analyze the plasma samples of study A and study B, respectively.

*LC-MS/MS method of study A*. In short, in study A, samples were deproteinized for 10 min at 4°C by a fivefold dilution in acetonitrile (ACN) containing internal standard (2 µM valyl-histidine). After centrifugation, supernatants were transferred to clean glass vials. Importantly, a further 1:2 dilution was performed to determine ANS in the samples withdrawn 60 min after ingesting 20 mg.kg^−1^ BM of CAR and ANS and 30 min after ingesting 30 mg.kg^−1^ BM CAR and ANS and a 1:5 dilution was performed on samples to measure ANS 60 min after ingesting 30 mg.kg^−1^ BM CAR and ANS. In case the obtained concentrations were outside of the calibration range (0 – 1.25 µM for CAR and 0 – 12.5 µM for ANS) due to a dilution factor that was too high or too low, the samples were re-analyzed with an adjusted dilution. Chromatographic separation was carried out with a Hypersil GOLD HILIC column (150 × 2.1 mm, particle size 3 µM, pore size 175 Å, Thermo Scientific) coupled with a Drop-In-Guard-Cartridge (5 µm). Ammonium formate in water (100 mM, pH 3 corrected with formic acid) and ACN were used as mobile phase A and B, respectively, at a flow rate of 0.250 mL.min^−1^. The method was qualified for CAR and ANS quantification in human EDTA plasma following the validation procedure reported by Matuszewski et al. (1998) to calculate limit of quantification, accuracy, and intermediate precision [25].

*LC-MS/MS method of study B*. In study B, 150 µL of plasma was added to 240 µL of methanol containing 1% formic acid and 10 µL internal standard (2.5 µM carnosine-D4 in ultrapure water). After centrifugation, 350 µL of supernatant was transferred to a 96-well plate and evaporated until a droplet (20 µL) remained. Thereafter, 90 µL of ultrapure water was added and vortexed. A calibration curve was made with a range of 0 to 15 µM for both CAR and ANS. In case concentrations were outside of the calibration range, samples were re-analyzed with a calibration curve ranging between 0 and 100 µM, with a 1:10 dilution. Chromatographic separation was achieved on an Acquity UPLC HSS T3 column (100 × 2.1 mm, dp: 1.8 µm) in combination with an Acquity HSS T3 1.8 µm Vanguard pre-column, both from Waters. Gradient elution was established with a mobile phase consisting of 0.1% (v/v) FA in water (Solvent A) and 0.1% (v/v) FA in ACN (Solvent B) at a flow rate of 0.4 mL.min^−1^.

### Serum carnosinase (CN1) activity

Serum samples of all subjects (Study A, B, and C) were collected throughout all three studies and stored at −20°C awaiting analysis. Serum CN1 activity was quantified by fluorometric determination of liberated histidine after CAR addition, the protocol was based on the one described by Teufel et al., 2003. The reaction was initiated by adding CAR (Flamma S.p.a., Chignolo d’Isola, Bergamo, Italy) to the serum and stopped after 10 min incubation at 37°C by adding 600 mM trichloroacetic acid (TCA). For controls, TCA was added before CAR. After centrifugation (15 min, 16,000 g), supernatant was added to a mixture of OPA (incomplete ophtaldehyde with 0.2% 2-mercaptoethanol) and 4 M sodium hydroxide and fluorescence was determined after 40 min (excitation: 360 nm and emission: 465 nm). All samples were analyzed in duplicate, the intra-assay coefficient of variability was 3.64% and inter-assay coefficient of variability was 10.41%.

### Calculations and statistical analysis

*Study A and B*. Mean and peak power relative to the participants’ body mass of each repeated sprint was calculated and the mean of these five values was used in the further analyses. The same method was applied for the peak torque during the three MVCs. Delta’s (difference between P30 and PRE (Study A) and between P60 and PRE (Study A and B)) of the sprints and MVCs were determined as a measure of improvement/deterioration compared to the PRE test. Shapiro-Wilk’s tests were used to control for normality of the data. Repeated measures (3 × 4 (A) and 2 × 2 (B)) ANOVA (time × condition) was used to analyze the mean and peak power (mean of five) and the peak torque (mean of three). A one-way ANOVA (study A) and a two-tailed paired samples t-test (study B) was used to check for differences in the calculated delta values. Post-hoc Tukey analyses were performed when appropriate. For both studies, HCD concentrations were compared between the time points and the conditions described.

Pearson correlations were conducted to reveal associations between CN1 activity of the subjects, HCD concentration in blood and the performance parameters. In case of identical circumstances (dose, timing, performance) between both studies, all subjects were taken together for these correlations. One subject participated in both studies, therefore, the mean value of the two studies, which showed similar results, was used for this subject.

*Study B*. The voluntary activation level (VAL) was calculated with the following equation: VAL% = 1 – (SIT amplitude/potentiated doublet amplitude) * 100. If the pulse of the SIT was not administered exactly during the maximal voluntary torque, a correction was applied using the following formula: VAL% = [1 – (SIT amplitude × voluntary torque level immediately before the SIT/maximal voluntary torque)/potentiated doublet amplitude] * 100. Repeated measures (2 × 2) ANOVA (time × condition) were used to analyze the VAL. Post-hoc Tukey analyses were performed when appropriate. Also, the potentiated stimuli during the MVC protocol were processed and the following parameters were analyzed: relative peak torque, time to maximal torque (time between the electrical pulse and the peak), contraction time (time between the beginning of the muscle contraction and the peak), half relaxation time and rate of force development (RFD) and the rate of force decline (RFD min). Moreover, the M wave duration and area under the curve were determined, measured in the vastus lateralis. Firstly, the mean of the three values was calculated followed by a calculation of the delta (difference between the P60 and PRE measurements) value. These delta values were then statistically analyzed: Two-tailed paired samples t-tests were conducted when data was normally distributed. In case of skewed data, Wilcoxon matched paired signed rank test was used for the analyses.

*Study C*. Incremental area under the curve (iAUC) was calculated with the trapezoidal method. Baseline blood flow was determined as the lowest measurement of three (10 minutes, 5 minutes and immediately before the HIT). Data on muscle blood flow and blood pressure were analyzed using repeated measures (17 × 3) ANOVA (time × condition). Post-hoc Tukey analyses were performed when appropriate. Effect of condition on iAUC was determined using a one-way ANOVA. Pearson correlations were used to check for associations between CN1 activity and other parameters.

*Study A, B, and C*. All data were statistically analyzed using GraphPad Prism (version 8; GraphPad Software, San Diego, California). A significance level of p ≤ 0.05 was used to identify statistical significance. Data are presented as mean ± SD or median ± IQR, for normally distributed and skewed data, respectively. Apart from p-values, 95% confidence intervals (95% CI) and effect sizes (ES) are shown.

## 3. RESULTS

### Plasma HCD

**Figure 2.**
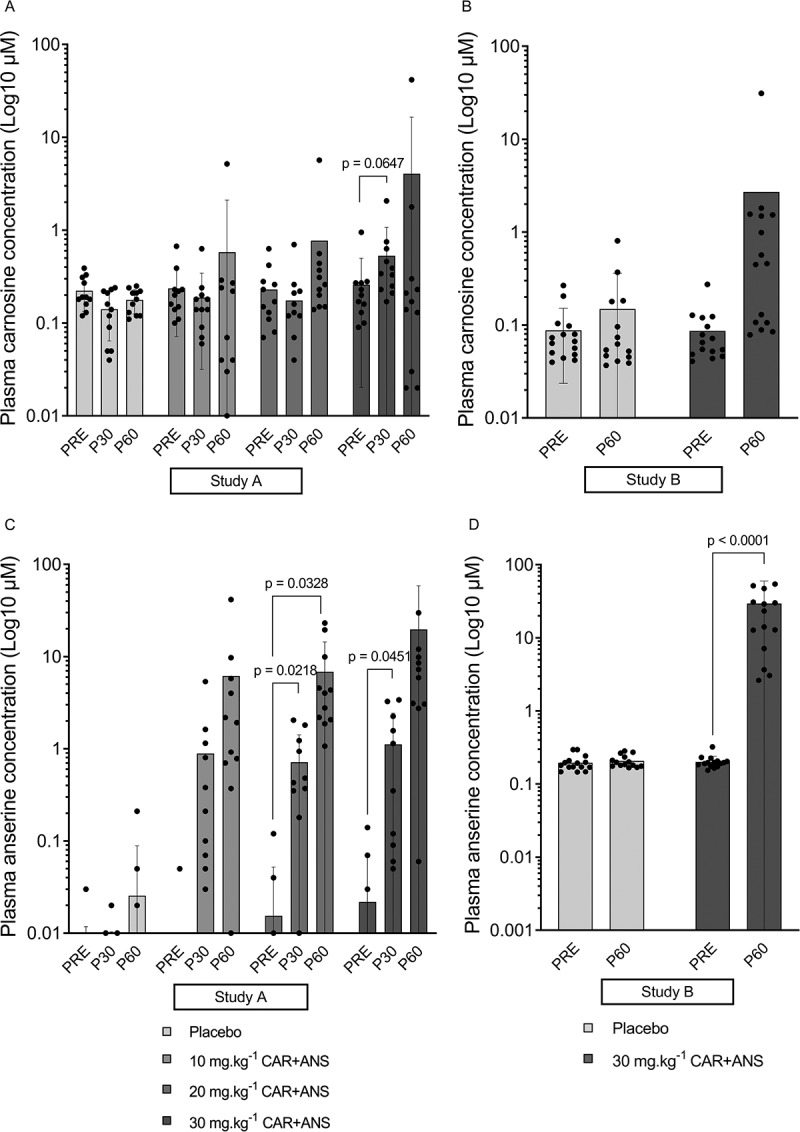
Log10 of the plasma carnosine concentration of study A (A) and B (B) and plasma anserine concentration of study A (C) and study B (D). Individual values, mean, SD and p-values in case of significance are shown.

*Study A*. Baseline plasma concentrations were in the range of 50 to 200 nM for CAR and undetectable to 200 nM for ANS, and did not change in the placebo intervention. Ingesting 10 or 20 mg.kg^−1^ of CAR + ANS did not significantly increase plasma CAR after 30 (P30) (p = 0.7076; 95% CI = −0.2119 to 0.1156; ES = 0.24 and p = 0.8047; 95% CI = to −0.2949 to 0.1840; ES = 0.17, respectively) or 60 minutes (P60) (p = 0.7324; 95% CI = −0.8872 to 1.573; ES = 0.23 and p = 0.5301; 95% CI = −0.7956 to 1.877; ES = 0.34, respectively). Following ingestion of 30 mg.kg^−1^, the CAR concentration increased twofold after 30 minutes (p = 0.0647; 95% CI = −0.0166 to 0.5584; ES = 0.78) and 16-fold after 60 minutes (p = 0.5876; 95% CI = −6.503 to 14.09; ES = 0.30) (Figure 2A). Plasma ANS levels show higher increases following ingestion: after ingesting 10 mg.kg^−1^, there was a non-significant 204-fold (p = 0.1953; 95% CI = −0.4060 to 2.171; ES = 0.55) and 1425-fold (p = 0.2545; 95% CI = −3.821 to 16.18; ES = 0.51) increase, after 30 and 60 minutes respectively. The 20 mg.kg^−1^ dose resulted in a concentration that was 45 times higher than baseline at P30 (p = 0.0218; 95% CI = −0.1087 to −1.286; ES = 0.98) and 433 times at P60 (p = 0.0328; 95% CI = −0.5851 to −13.08; ES = 0.90). Lastly, after ingesting 30 mg.kg^−1^ of CAR + ANS, the ANS concentration was 50-fold higher (p = 0.0451; 95% CI = −0.0247 to 2.164; ES = 0.85) after 30 minutes (P30) and 885-fold (p = 0.2599; 95% CI = −12.46 to 51.86; ES = 0.51) after 60 minutes (P60) compared to baseline. The fact that some pronounced fold-changes were not significant can be ascribed to large inter-individual differences in response to supplementation. Some subjects show a very large increase while others only show moderate or small increases (Figure 2C).

*Study B*. Although not significant, the CAR concentration increased 31-fold (p = 0.1576; 95% CI = −0.816 to 6.058; ES = 0.33) at P60 compared to baseline following 30 mg.kg^−1^ CAR + ANS. The ANS concentration was 146 times higher (p < 0.0001; 95% CI = −42.49 to −16.03; ES = 0.96) after ingesting 30 mg.kg^−1^ of CAR + ANS (Figure 2B, D). See supplementary table 1 for detailed concentrations of plasma carnosine and anserine.

**Figure 3.**
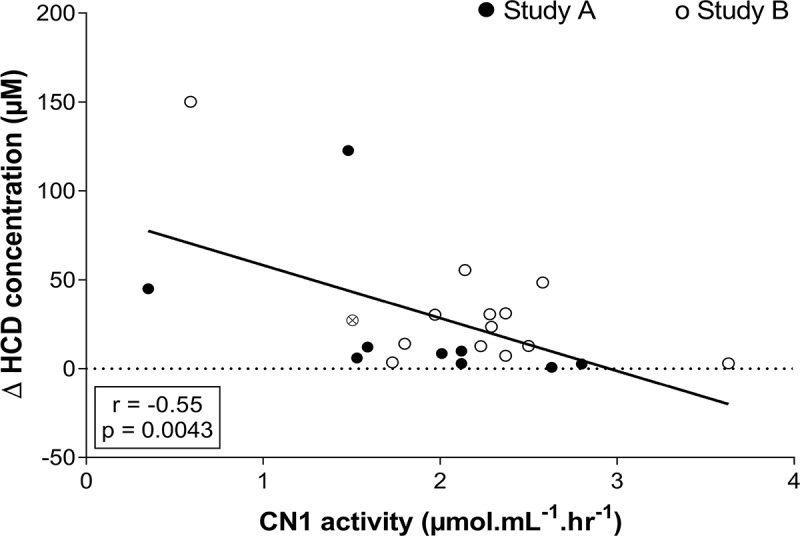
Pearson correlation between delta (P60 – PRE) plasma HCD concentration and CN1 activity after ingesting 30 mg.kg-1 CAR + ANS. Filled circles and empty circles show the individual values of the subjects in study A and study B, respectively. The gray circle indicates the mean value of the subject who participated in both studies.

*Study A and B*. A correlation was made between the delta (difference between P60 and PRE) plasma HCD concentration and the CN1 activity of both studies together in the conditions that were identical in timing and dose ingested. A negative correlation was found between CN1 activity and delta HCD concentration at P60 after ingesting 30 mg.kg^−1^ CAR + ANS (r = −0.55; p = 0.0043; 95% CI = −0.78 to −0.20) (Figure 3).

For the remaining conditions in study A, negative correlations were found between CN1 activity and delta plasma HCD at P30 (r = −0.82; p = 0.0015; 95% CI = −0.95 to −0.42) and P60 (r = −0.88; p = 0.0004; 95% CI = −0.97 to −0.58) following the ingestion of 10 mg.kg^−1^ CAR + ANS and between CN1 activity and delta plasma HCD at P60 after ingesting 20 mg.kg^−1^ CAR + ANS (r = −0.81; p = 0.0027; 95% CI = −0.95 to −0.40), indicating that a lower CN1 activity promotes higher HCD concentrations in plasma after supplementation.

### Performance parameters

Several performance parameters in both study A and B were analyzed to investigate whether the supplement affected MVC and sprint performance.

**Figure 4.**
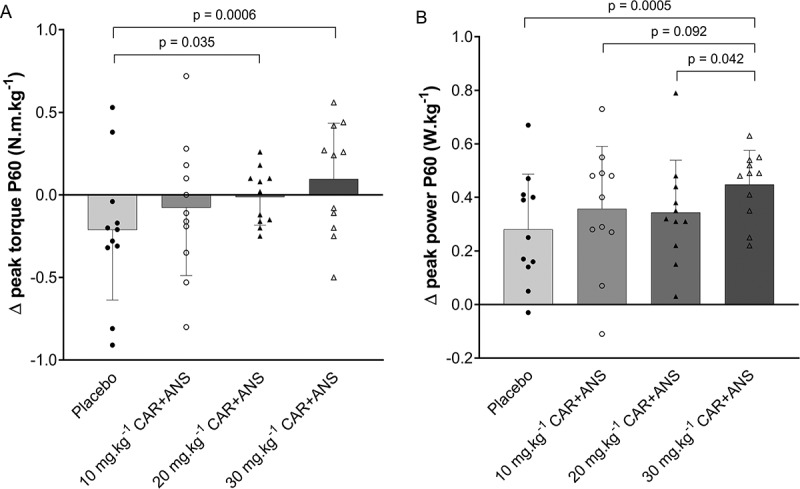
Performance parameters of study A: (A) shows the delta (P60 – PRE) peak torque values of the MVCs and (B) shows the delta (P60 – PRE) peak power of the RSA test. Positive values indicate an improvement of performance from pre to P60, negative values indicate an attenuation of performance from pre to P60. Individual values, mean, SD and p-values in case of significance are shown.

*Study A*. No significant interaction effect (p = 0.11, ES = 0.16) was found for MVC peak torque. To account for day-to-day variability in performance, delta peak torque values (difference between P30 and PRE and between P60 and PRE) were analyzed. A trend to a significant interaction effect was found (time × condition: p = 0.085; ES = 0.20). The change in performance did not differ between conditions at P30 while at P60, a significant higher delta peak torque was found for the 20 mg.kg^−1^ CAR + ANS (p = 0.03; 95% CI = 0.01 to 0.39; ES = 0.55) and 30 mg.kg^−1^ CAR + ANS (p = 0.0006; 95% CI = 0.12 to 0.50; ES = 0.87) compared to the placebo condition (Figure 4A). In the placebo condition, the MVC performance was attenuated throughout successive measurements on one test day (−4.41 ± 11.2% compared to PRE), as illustrated by the negative delta values, while a stepwise counteraction of this loss in force occurs with increasing doses. Individual values, means, and SD of these delta values at P60 vs PRE are depicted in Figure 4A.

Mean and peak power of the repeated cycling sprints demonstrated a significant improvement over time within each test day (main effect time: p < 0.0001; ES = 0.86 for mean power; main effect of time: p < 0.0001; ES = 0.86 for peak power). Mean power at P30 was 2.61 ± 1.52% higher compared to PRE (p < 0.0001; 95% CI = 0.18 to 0.32; ES = 2.44) and 3.20 ± 1.73% at P60 compared to PRE (p < 0.0001; 95% CI = 0.23 to 0.38; ES = 2.64). Similarly, for peak power, subjects performed on average 2.72 ± 1.60% better at P30 (p < 0.0001; 95% CI = 0.21 to 0.38; ES = 2.48) and 3.29 ± 1.80% at P60 (p < 0.0001; 95% CI = 0.27 to 0.44; ES = 2.60) compared to PRE. When considering delta mean power, significant interaction effect was observed (time × condition: p = 0.038; ES = 0.24). An improvement was found for the 30 mg.kg^−1^ CAR + ANS condition at P60 compared to the placebo (p = 0.0094; 95% CI = 0.277 to 0.456; ES = 0.64) while none of the other conditions showed improvements. When looking into delta peak power values (difference between P30 and PRE and between P60 and PRE), no significant interaction was observed (p = 0.11; ES = 0.18). However, because of the interest in the separate doses and time points, each condition was looked into separately. At P30 no important performance improvements were found for delta peak power while at P60 however (Figure 4B), a higher delta peak power was found when 30 mg.kg^−1^ CAR + ANS was ingested compared to placebo (p = 0.0005; 95% CI = 0.07 to 0.27; ES = 0.91), 10 mg.kg^−1^ CAR + ANS (p = 0.09; 95% CI = 0.01 to 0.19; ES = 0.27) and 20 mg.kg^−1^ CAR + ANS (p = 0.04; 95% CI = 0.01 to 0.21; ES = 0.24). Supplementary table 2 shows detailed results of mean power, peak power, and peak torque of the different conditions and time points.

*Study B*. No significant interaction effect (time × condition: p = 0.22; ES = 0.11), on MVC peak torque was found. Delta peak torque values (difference between P60 and PRE) did not differ between conditions (p = 0.68; 95% CI = −0.16 to 0.21; ES = 0.11), as opposed to what was found in study A.

Regarding the mean power of the RSA, similar to study A, subjects performed on average 2.20 ± 1.67% better at P60 compared to PRE (main effect of time: p < 0.0001; 95% CI = 0.13 to 0.26; ES = 0.52). Also, the subjects reached a 2.32 ± 1.70% higher peak power at P60 compared to the PRE measurement (main effect of time: p = 0.0003; 95% CI = 0.11 to 0.28; ES = 0.61). When considering the delta values (difference between P60 and PRE), no effect of the supplement was found, not for mean power (p = 0.86; 95% CI = −0.08 to 0.10; ES = 0.17), nor for peak power (p = 0.56; 95% CI = −0.12 to 0.07; ES = 0.17). Supplementary table 2 shows detailed results of mean power, peak power, and peak torque of the different conditions and time points.

**Figure 5.**
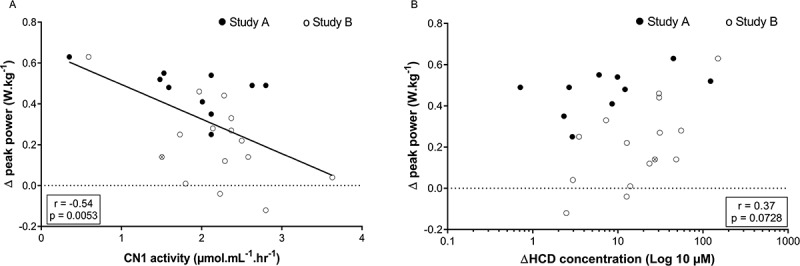
Pearson correlation between (A) the CN1 activity of all subjects or (B) the delta plasma HCD concentration and their delta peak power in the RSA test after ingesting 30 mg.kg^−1^ CAR + ANS. Filled circles and empty circles show the individual values of the subjects in study A and study B, respectively. The gray circle indicates the mean value of the subject who participated in both studies.

*Combined results of study A and B*. Performance results of study A suggest that a higher dose, and thus higher plasma HCD concentration, is needed to elicit an ergogenic effect. Hence, it is possible that people with a lower CN1 activity, and higher increase in plasma HCD concentrations after supplementation, are favored. After ingesting 30 mg.kg^−1^ CAR + ANS, no correlation was found between MVC performance and CN1 activity (r = 0.11; p = 0.59; 95% CI = −0.49 to 0.29), nor with delta HCD concentration (r = −0.23; p = 0.26; 95% CI = −0.17 to 0.58). For the RSA performance, on the other hand, a significant negative correlation was found with CN1 activity (r = −0.54; p = 0.0053; 95% CI = 0.19 to 0.77) (Figure 5A) and the positive correlation with delta HCD concentration tended toward significance (r = 0.37; p = 0.0728; 95% CI = −0.66 to 0.03; Figure 5B). This suggests a greater cycling sprint performance improvement with lower CN1 activity and, as a result, higher HCD increase in plasma.

### Underlying mechanism

*Study B*. MVCs were performed with an electrical stimulation protocol to explore an effect on neuromuscular function as a potential ergogenic mechanism. On the placebo day, the voluntary activation level (VAL) was on average 80.28 ± 8.05% and 79.86 ± 8.74% at PRE and P60, respectively. The VAL on the 30 mg.kg^−1^ CAR + ANS day was 80.92 ± 8.83% at PRE and 80.54 ± 10.41% at P60. No differences were found (interaction effect (condition × time): p = 0.92; 95% CI = −3.11 to 2.82; ES = 0.001), indicating that ingestion of CAR + ANS had no influence on the VAL of the subjects.

For the twitches administered following the MVC, no differences were found between the placebo condition and the HCD condition for the maximal peak torque, time to maximal torque, contraction time, rate of force development (RFD), rate of force decline (RFD min), half relaxation time and the M wave parameters. Table 1 shows the mean/median ± SD/IQR of the delta’s and their corresponding p values of the singlet. Parameters of the 10 Hz and 100 Hz doublets are not shown, however, similar results were found.

**Table 1.** Overview of the parameters calculated for the singlet in the MVC test (study B)

		PLACEBO					30 mg.kg^−1^ CAR + ANS						
		Normally distributed?	Mean/median PRE	SD/IQR	Mean/median P60	SD/IQR	Mean/median PRE	SD/IQR	Mean/median P60	SD/IQR	95% CI	Effect size	P-value Significant?
Singlet	MAX (N.m)	No	57.2	11.4	55.8	12.1	56.6	14.6	56.4	14.5	−0.10 to 0.04	0.03	0.93 NS
	Time to max (ms)	Yes	110	16	110	17	109	17	109	19	−7.56 to 6.63	0.04	0.89 NS
	Contraction time (ms)	Yes	57	17	57	18	56	18	55	18	−10.48 to 8.46	0.06	0.82 NS
	Half relaxation time (ms)	Yes	78	11	80	14	79	10	79	13	−7.61 to 5.27	0.10	0.70 NS
	RFD (N.m.s^−1^)	No	1626	266	1553	296	1505	366	1409	378	−128.9 to 147.4	0.07	0.80 NS
	RFD min (N.m.s^−1^)	No	661	142	611	138	587	199	580	237	−116.80 to 20.38	0.39	0.14 NS

Rate of force development (RFD), rate of force decline (RFD min). Mean/median values of each time point and condition are reported. If the data is normally distributed, mean ± SD values are reported, in case of skewed data, median ± IQR is shown.

**Figure 6.**
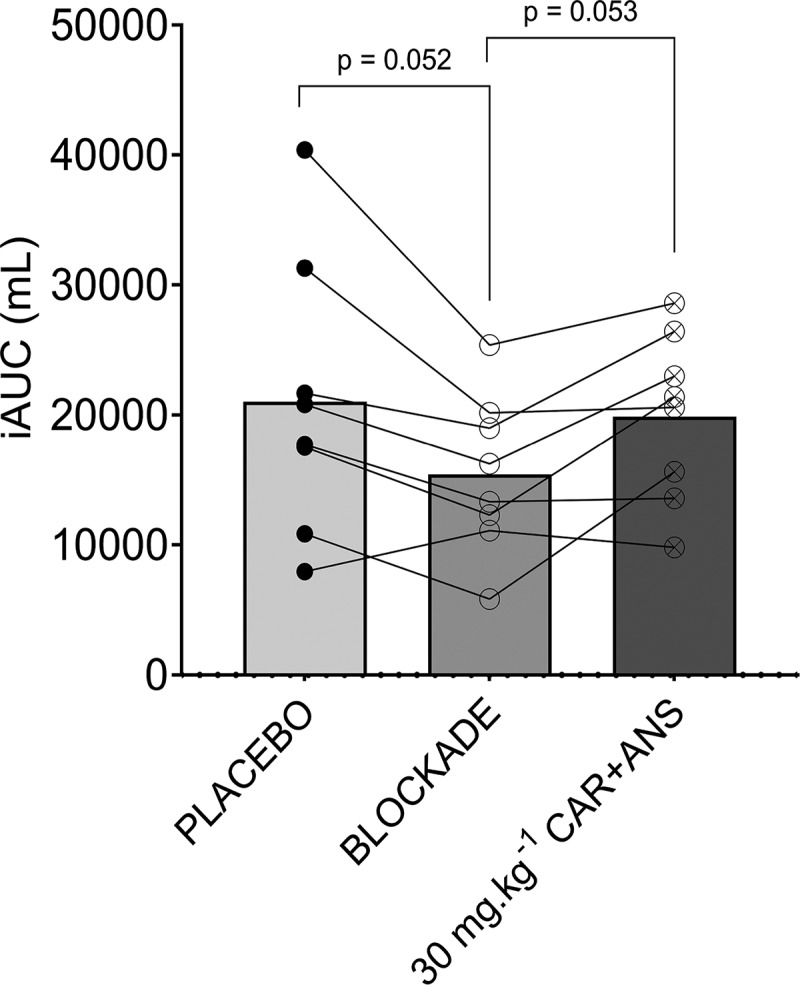
iAUC of the placebo, blockade and 30 mg.kg^−1^ CAR + ANS condition. Individual values, mean bars and p-values are shown.

*Study C*. Post-exercise blood flow was monitored to examine whether the supplement affected performance through improved muscle recovery. Baseline blood flow did not differ between the test days (p = 0.42; ES = 0.11). Also, peak blood flow post-exercise did not differ between the conditions (p = 0.58; ES = 0.09), indicating that the supplements have no effect on muscle perfusion immediately after the exercise. When calculating the degree of blood flow elevation above baseline for the 120 min post-exercise period, a significant effect of the condition (p = 0.0455; ES = 0.38) was found. A trend to a significantly lower iAUC was found for the BLOCKADE compared to the placebo (−27%; p = 0.052; 95% CI = −48.30 to 11,282). The iAUC after taking the placebo and CAR + ANS condition did not differ (p = 0.88; 95% CI = −5974 to 8292) (Figure 6).

Other parameters related to post-exercise hyperemia were compared. No differences between the conditions were found for mean arterial pressure (Time × Condition: p = 0.72; ES = 0.08) and vascular conductance (Time × Condition: p = 0.41; ES = 0.13). In all three conditions, the mean arterial pressure increased toward the end of the recovery (Main effect of time: p = 0.0081; ES = 0.41).

## 4. DISCUSSION

Three studies were conducted to first define the optimal dose of acute CAR and ANS ingestion to improve performance, and secondly to explore potential mechanisms underlying the observed ergogenic effects.

The data from the dose-response study (Study A) provide evidence that acute pre-exercise ingestion of combined CAR and ANS could potentially enhance certain aspects of short maximal exercise performances, such as the peak power and torque during short cycling sprints and maximal contractions. Stepwise dose-dependent ergogenic effects were detected for maximal voluntary isometric knee extension torque and for mean and peak power on repeated (5x 6 s with 24 s recovery) maximal cycling sprints. While no effect was observed with the 10 mg.kg^−1^ BM dose, a moderate effect was seen for the 20 mg.kg^−1^ BM and a significant effect was obtained following the 30 mg.kg^−1^ BM dose for both the knee torque and cycling sprint parameters. The currently used dosages consist of a 1:1 ratio (e.g. 30 mg.kg^−1^ BM of CAR + 30 mg.kg^−1^ BM of ANS), so the minimal effective total dose of HCD is in the range of 40 to 60 mg.kg^−1^ BM. This is in line with Blancquaert et al. (2021), who found an ergogenic effect from a 50 mg.kg^−1^ BM dose (1:1 ratio for CAR:ANS in pure form) and Barbaresi et al. (2021) who used 45 mg.kg^−1^ BM (1:2 ratio for CAR:ANS in chicken broth form). Suzuki et al. (2006) used a fixed dose, so not body mass corrected, which would correspond to 23 mg.kg^−1^ BM for a 1:3 CAR:ANS mixture. This lower dose compared to the studies of Blancquaert et al. (2021) and Barbaresi et al. (2021) may explain their mixed results.

In addition to defining the minimal effective dose for acute CAR and ANS ingestion, which from the collectively available studies seems to lie around 40–60 mg.kg^−1^ BM, also the composition of the dipeptides mixture may be important. Initial studies were performed with pure CAR, but dosages around 10–20 mg.kg^−1^ BM do not elicit any appreciable elevation of plasma CAR concentration and were not ergogenic [7]. Higher doses up to 60 mg.kg^−1^ BM are still only partially effective in inducing carnosinemia, and can be prone to side-effects like headache [11,26] and were therefore not tested for exercise performance. Addition of ANS to the CAR supplement seems more preferable, as ANS is more resistant to degradation by CN1 [10] and additionally stabilizes co-ingested CAR by competitive inhibition to CN1 [12,13]. The currently used ratio of 1:1 CAR:ANS seems to be a valid ergogenic approach. The more natural ratio of CAR to ANS in chicken meat, 1:2 to 1:3 (depending on meat source) may also work for acute pre-exercise supplementation [15,16]. Finally, whether pure ANS is effective, remains to be established.

Two lines of evidence support the rationale that the level of elevation of circulating plasma levels of CAR and ANS is a key defining parameter in yielding an ergogenic effect. First, the dose-response study showed that performance enhancement was obtained 60 minutes, but not 30 minutes post ingestion. This is fully in line with the plasma HCD levels being only marginally elevated (< 2 µM total HCD) at 30 min, but more strongly elevated (>20 µM total HCD) at 60 minutes. Second, there was an overall significant negative correlation between CN1 activity and performance improvements, and a tendency for a positive correlation between plasma HCD increase and performance enhancement, suggesting elevated plasma HCDs as a prerequisite to elicit an ergogenic effect.

When combining the obtained information from the current study and existing literature for the different doses, the timing and the CN1 responsiveness, a minimal threshold for ergogenic effectiveness with respect to ingested dose is estimated to be 40–60 mg.kg^−1^ BM of a 1:1 to 1:3 ratio of CAR:ANS ingested 60 minutes before exercise. With respect to minimal effectiveness of resulting plasma HCD levels, based on the results from Blancquaert et al. (2021) and Barbaresi et al. (2021), a concentration of 15–20 µM is estimated. However, evaluation of the individual results of our studies did not result in a straightforward conclusion. Some subjects with lower HCD concentrations improved more than subjects with higher concentrations, especially when looking at the MVC data. The cycling sprint data were clearer. Especially in study B, where the person with the highest concentration had by far the largest effect of the supplement.

Although these collective findings tend to make sense, it is unclear why we did not observe an ergogenic effect in study B, in which we adopted this minimal effective dose and strategy described above. Part of the explanation may lie in the diversity in CN1 activity, because when combining study A and B (Figure 6), or when looking at study B alone (r = −0.6764 and p = 0.0056), we found a negative correlation between CN1 activity and performance improvement. This indicates that there was some degree of ergogenicity in people with low CN1 activity in study B, which was not significant at the whole group level. The mean CN1 activity in study B was higher than in study A, 2.2 ± 0.7 µmol.mL^−1^.hr^−1^ compared to 1.9 ± 0.7 µmol.mL^−1^.hr^−1^, respectively. This could indicate that people with lower CN1 activity, and higher plasma CAR and ANS, are more likely to benefit from the supplement [27].

Another part of the explanation for not finding ergogenic effects in study B, may lie in the differences in study design. In study A, the subjects performed three exercise test sessions with only 30 minutes between sessions, while in study B, they performed only 2 test sessions with 90 minutes recovery. Possibly, the subjects received sufficient time to recover in study B while in study A, subjects who received the HCD as supplement, had an improved recovery due to higher muscle perfusion during those 30 minutes. Borne and colleagues (2017) provided evidence that mechanically (through low-frequency neuromuscular electrostimulation) increasing muscle blood flow between repeated bouts of intense exercise improves recovery and performance [28]. A potential hemodynamic influence was therefore explored in study C.

CAR has been shown to positively affect muscle blood flow either by a direct effect on blood vessel relaxation [29] or indirectly through autonomic innervation [18,19]. CAR can induce vascular relaxation through a non-endothelial-dependent cyclic GMP mechanism [29], albeit in concentrations that were substantially higher (625 µM – 20,000 µM) than concentrations reached through supplementation in our study. These direct or indirect vascular effects could be mediated by histaminergic signaling, as suggested by Boldyrev et al in 2013, based on earlier findings in rats pointing toward CAR as a histidine reservoir for the production of histamine [30]. Our data, however, do not support such mechanism. We did find a 27% decrease in muscle perfusion after ingesting a histamine receptor blockade, which was in line with previous findings [23,24], indicating that histaminergic signaling is important in post-exercise muscle perfusion. However, should CAR serve as a histamine donor, an increase in muscle perfusion after ingesting the supplement could be expected. Yet, post-exercise hyperemia remained unchanged after ingesting 30 mg.kg^−1^ of both CAR and ANS. These results indicate that the HCD did not affect muscle blood flow in the current experimental set-up, and could not explain the absence of ergogenic effect in study B. Importantly, it should be mentioned that all subjects in these studies were healthy, physically active, young men. The absence of an effect on muscle blood flow could be explained by the fact that there is no room for improvement in this system. It might be interesting to look at effects of CAR and ANS on muscle perfusion in people with an impaired vascular system.

In study B, we looked into effects on neuromuscular function that could be responsible for the observed performance improvements. This idea originated from the Severin’s Phenomenon. Herein, an immediate recovery from muscle fatigue is observed, with an increase in muscle contraction force and time to exhaustion when CAR is added to a neuromuscular preparation of a frog muscle [4]. This experiment, along with findings in which CAR affects the sympathetic nerve activity was the basis for our study B. The sympathetic nervous system is involved in multiple functions of the skeletal muscle cell such as the ionic fluxes through the membrane, acetylcholine release from the motor end plate and Ca^2+^ release and reuptake in the sarcoplasmic reticulum [31]. More recent findings show that innervation is crucial for the synaptic integrity and function of the neuromuscular junctions [32]. This could indicate that ingestion of the HCD affects the autonomic nervous system and through this improves performance. In rats, topically administering 10 pg CAR in 0.1 mL saline (which corresponds to 0.000442 µM) increased sympathetic nerve activity in the contralateral skeletal muscle [19]. With a mean concentration of 2.7 µM of CAR and 29.5 µM ANS in plasma after administering 30 mg.kg^−1^ of both, we observed no changes in voluntary and electrically evoked contraction parameters. It is known that exercise induces an increase in vascular permeability [33], potentially making it possible for CAR and ANS to enter the interstitial space between blood vessels and muscle. This would resemble the situation of topical application of CAR on muscle or the addition of CAR in the solution used in the Severin experiment. Thus, despite not seeing a group-level ergogenic effect in this study, a direct effect on neuromuscular junctions seems unlikely.

These experiments had certain limitations. In study A, no higher doses were tested than up to 30 mg.kg^−1^ of CAR and ANS. It could be argumented that higher doses should be tested as well to see whether performances can be improved even further. However, apart from a risk of side-effects such as paresthesia and headaches with higher doses, it was also found by Nagai et al. (2019) that too high doses abolish the effects of the HCD. Secondly, no measurements were performed beyond 60 minutes post supplementation. This time frame was based on the pharmacokinetic profile, revealing a peak in CAR and ANS 60 minutes after ingestion. Our data indicate that performance improvements are related to higher intact HCD concentrations, hence waiting any longer could reduce intact HCDs in the blood stream due to the highly active CN1 enzyme. Additionally, in study C, Doppler Ultrasound was used to measure muscle perfusion. The coefficient of variance (CV%) between the baseline measurements on the three test days was 13%. Previous research in rats show a 5–15% increase in muscle blood flow, measured directly on the muscle [18,19]. The relatively high CV%, in combination with small effects on blood flow, could be an explanation for why we were not able to pick up differences between the placebo and supplemented conditions.

## 5. CONCLUSIONS

To summarize, in line with recent studies from our lab, the current study reveals that oral acute ingestion of 20 and certainly 30 mg.kg^−1^ body mass of each CAR and ANS, 60 minutes before a high intensity exercise, can potentially improve muscle and exercise performance. People with lower plasma CN1 activity and thus a slower breakdown of circulating CAR and ANS, tend to benefit more from this ergogenic effect compared to people with a higher enzyme activity. Finally, neither the involvement of a direct effect on neuromuscular function, nor an indirect effect on recovery through increased muscle perfusion, as a potential mechanism of action could be confirmed in this study.

## Supplementary Material

Supplemental Material
