# Supplementary material for: The ergogenic effect of acute carnosine and anserine supplementation: dosing, timing, and underlying mechanism
Source: J Int Soc Sports Nutr. 2022 Mar 26;19(1):70–91. doi: 10.1080/15502783.2022.2053300 (PMC9116398; doi:10.1080/15502783.2022.2053300)
Supplement: Supplemental Material [file RSSN_A_2053300_SM2604.docx]

# Supplementary material of the manuscript: The ergogenic effect of acute carnosine and anserine supplementation: dosing, timing and underlying mechanism

Breakfast study A: On the actual test days, subjects arrived in a fasted state and received a standardized breakfast free of CAR and ANS immediately upon arrival. The breakfast consisted of the following components: plain white bread, strawberry jam, white yoghurt and a banana. The first test day, subjects were free to eat what and how much they liked. The following test days, they had to eat exactly the same.

Breakfast study B: On the actual test days, subjects arrived in a fasted state and received a standardized breakfast free of CAR and ANS after the electrodes were put in place. The breakfast consisted of the following components: White bread, strawberry jam, 150 mL of semi-skimmed milk and a banana. The amount of bread and jam was calculated to reach a total of 2g of carbohydrates per kg.

*Supplementary table 1 – Plasma carnosine and anserine concentration of study A and B*

|  |  | **STUDY A** | | | |
| --- | --- | --- | --- | --- | --- |
|  |  | **CAR (µM)** | | **ANS (µM)** | |
|  |  | **Mean** | **SD** | **Mean** | **SD** |
| **PLACEBO** | **PRE** | 0,22 | 0,08 | 0 | 0,01 |
|  | **P30** | 0,14 | 0,07 | 0 | 0 |
|  | **P60** | 0,18 | 0,05 | 0,03 | 0,06 |
| **10 mg.kg-1 CAR+ANS** | **PRE** | 0,24 | 0,16 | 0 | 0,03 |
|  | **P30** | 0,19 | 0,15 | 0,89 | 0,7 |
|  | **P60** | 0,58 | 1,52 | 6,19 | 12,12 |
| **20 mg.kg-1 CAR+ANS** | **PRE** | 0,23 | 0,16 | 0,02 | 0,03 |
|  | **P30** | 0,19 | 0,18 | 0,71* | 0,7 |
|  | **P60** | 0,77 | 1,62 | 6,85* | 7,55 |
| **30 mg.kg-1 CAR+ANS** | **PRE** | 0,26 | 0,23 | 0,05 | 0,04 |
|  | **P30** | 0,53 | 0,54 | 1,12* | 1,31 |
|  | **P60** | 4,05 | 12,44 | 19,72 | 38,95 |
|  |  | **STUDY B** | | | |
| **PLACEBO** | **PRE** | 0,09 | 0,06 | 0,196 | 0,05 |
|  | **P60** | 0,14 | 0,2 | 0,194 | 0,1 |
| **30 mg.kg-1 CAR+ANS** | **PRE** | 0,09 | 0,06 | 0,201 | 0,04 |
|  | **P60** | 2,71 | 7,9 | 29,46* | 30,4 |

*p < 0.05 compared to the PRE value

*Supplementary table 2 – Results of the RSA (mean and peak power) and MVC (peak torque) of the different conditions and time points of both study A and B*

|  |  | **STUDY A** | | | | | |
| --- | --- | --- | --- | --- | --- | --- | --- |
|  |  | **Mean power (W.kg^-1^)** | | **Peak power (W.kg^-1^)** | | **Peak torque (N.m.kg^-1^)** | |
|  |  | **Mean** | **SD** | **Mean** | **SD** | **Mean** | **SD** |
| **PLACEBO** | **PRE** | 9,54 | 0,40 | 10,84 | 0,49 | 3,68 | 0,98 |
|  | **P30** | 9,82 | 0,45 | 11,13 | 0,55 | 3,68 | 0,93 |
|  | **P60** | 9,79 | 0,41 | 11,12 | 0,51 | 3,47 | 0,79 |
| **10 mg.kg-1 CAR+ANS** | **PRE** | 9,54 | 0,39 | 10,80 | 0,43 | 3,67 | 0,90 |
|  | **P30** | 9,78 | 0,46 | 11,09 | 0,53 | 3,71 | 0,74 |
|  | **P60** | 9,85 | 0,53 | 11,15 | 0,61 | 3,59 | 0,77 |
| **20 mg.kg-1 CAR+ANS** | **PRE** | 9,52 | 0,43 | 10,78 | 0,51 | 3,48 | 0,73 |
|  | **P30** | 9,72 | 0,53 | 11,00 | 0,62 | 3,54 | 0,70 |
|  | **P60** | 9,82 | 0,52 | 11,12 | 0,63 | 3,47 | 0,75 |
| **30 mg.kg-1 CAR+ANS** | **PRE** | 9,53 | 0,54 | 10,77 | 0,59 | 3,50 | 0,93 |
|  | **P30** | 9,81 | 0,58 | 11,14 | 0,62 | 3,55 | 0,81 |
|  | **P60** | 9,89 | 0,59 | 11,22 | 0,64 | 3,60 | 0,74 |
|  |  | **STUDY B** | | | | | |
| **PLACEBO** | **PRE** | 8,85 | 0,41 | 10,04 | 0,46 | 3,35 | 0,66 |
|  | **P60** | 9,07 | 0,41 | 10,27 | 0,49 | 3,36 | 0,61 |
| **30 mg.kg-1 CAR+ANS** | **PRE** | 8,85 | 0,48 | 10,01 | 0,56 | 3,35 | 0,73 |
|  | **P60** | 9,02 | 0,48 | 10,25 | 0,56 | 3,32 | 0,79 |
